# Rheumatic heart disease: infectious disease origin, chronic care approach

**DOI:** 10.1186/s12913-017-2747-5

**Published:** 2017-11-29

**Authors:** Judith M Katzenellenbogen, Anna P Ralph, Rosemary Wyber, Jonathan R Carapetis

**Affiliations:** 1 Telethon Kids Institute, The University of Western Australia, Perth, Western Australia; 2 School of Population and Global Health, The University of Western Australia, Perth, Western Australia; 3 Global and Tropical Health, Menzies School of Health Research, Darwin, Northern Territory Australia; 4 Division of Medicine, Royal Darwin Hospital, Darwin, NT Australia; 5 Princess Margaret Hospital for Children, Perth, Western Australia

**Keywords:** Acute rheumatic fever, Rheumatic heart disease, Prevention, Chronic care

## Abstract

**Background:**

Rheumatic heart disease (RHD) is a chronic cardiac condition with an infectious aetiology, causing high disease burden in low-income settings. Affected individuals are young and associated morbidity is high. However, RHD is relatively neglected due to the populations involved and its lower incidence relative to other heart diseases.

**Methods and results:**

In this narrative review, we describe how RHD care can be informed by and integrated with models of care developed for priority non-communicable diseases (coronary heart disease), and high-burden communicable diseases (tuberculosis). Examining the four-level prevention model (primordial through tertiary prevention) suggests primordial and primary prevention of RHD can leverage off existing tuberculosis control efforts, given shared risk factors. Successes in coronary heart disease control provide inspiration for similarly bold initiatives for RHD. Further, we illustrate how the Chronic Care Model (CCM), developed for use in non-communicable diseases, offers a relevant framework to approach RHD care. Systems strengthening through greater integration of services can improve RHD programs.

**Conclusion:**

Strengthening of systems through integration/linkages with other well-performing and resourced services in conjunction with policies to adopt the CCM framework for the secondary and tertiary prevention of RHD in settings with limited resources, has the potential to significantly reduce the burden of RHD globally. More research is required to provide evidence-based recommendations for policy and service design.

## Background

The epidemiological transition - the complex changes in patterns of disease in populations from a dominance of infectious disease to non-communicable disease (NCD) [1] - has had an enormous effect on the burden of disease and nature of health care in high income countries (HICs). In low and middle income countries (LMICs) the high burden of infectious disease and NCD coincide, continuing to challenge the management of disease and healthcare resourcing [2]. Globally, infectious disease and NCD epidemics are converging in terms of disease complexity, dual causality and demand on services [3].

Rheumatic heart disease (RHD) is characterised by permanent damage to the valves of the heart that develops as a serious consequence of repeated episodes of acute rheumatic fever (ARF), an autoimmune reaction to a Group A streptococcus (GAS) bacterial infection. Heart failure, atrial fibrillation and stroke are common complications of RHD, resulting in significant premature morbidity and mortality. In HICs RHD is now rare although persisting in at-risk population subgroups. However, the epicentre of RHD has shifted to LMICs, and advances in treatment, research and prevention have moved to these countries [4, 5]. The global focus on RHD is expected to increase as a result of the recommendation made by Executive Board of the World Health Organization in June 2017 for prioritisation of a Rheumatic Fever and Rheumatic Heart Disease Prevention and Control Strategy for adoption at the 2018 World Health Assembly [6]. Yet there remains a lack of clarity about how to implement proven methods of controlling RHD in different settings. Traditionally, approaches have been built around a relatively narrow infectious diseases perspective, based on GAS infection as the root cause of ARF. However, in contemporary endemic settings, improvements in diagnosis and management of RHD require a shift to a broader chronic disease model of care. Therefore, ARF and RHD represent a classic example of how infectious disease and NCD approaches converge.

This review aims to demonstrate how health services for the prevention and control of RHD can be informed by both infectious and chronic disease models. Specifically, RHD control approaches are compared and contrasted with a classic NCD, coronary heart disease (CHD), and a classic infectious disease, tuberculosis (TB) using a levels of prevention framework approach [7]. We outline which prevention components of each disease require a medium to long-term disease management approach exemplified by the Chronic Care Model [8, 9] and further discuss how synergies might be developed to strengthen the different programs. A unified global approach to managing chronic disease is timely as globalization shifts diseases of development to LMICs and as refugees and migrants with diseases of poverty move to developed settings.

## Methods

This is a narrative review [10, 11] the aim of which is to provide an up-to-date overview of health system approaches for the prevention and control of RHD in high-burden settings. We describe how RHD control approaches are placed in the context of the classic disease prevention framework, drawing on the TB and CHD literature for comparative purposes. Sources of information to inform the review were derived from searches of PubMed, manual searches of the references of retrieved literature, personal libraries and experience in delivering RHD, tuberculosis and coronary heart disease care in high-burden settings. The paper provides an overview of RHD aetiology and epidemiology to provide the required context, and describes control approaches applicable at the levels of primordial, primary, secondary and tertiary prevention. It then examines the application of vertical, horizontal and diagonal control programs. Lessons that can be drawn from both infectious and chronic disease models of care are provided, and how these may be applied in the health system context.

## Results - Review Topics

### RHD: Disease development and progression

RHD begins with a GAS infection of the pharynx, or potentially the skin [12, 13] with ARF developing in a small minority of susceptible cases resulting from an abnormal immune response to GAS infection. Risk of developing ARF is a function of bacterial, genetic and environmental factors. The importance of GAS strain type remains relatively poorly understood [14, 15]. Understanding the genetic susceptibility to ARF/RHD is the subject of active research [16, 17] with estimates of ARF heritability estimated to be as high as 60% in a meta-analysis of twin studies [18].

Environmental conditions appear to be the major determinant of ARF and RHD; settings which promote high GAS exposure are associated with socioeconomic deprivation. The most consistent determinant of ARF incidence is overcrowding, exemplified in United States military barrack studies in the 1950s [19]. The importance of overcrowding is also recognized in contemporary studies [20] along with other environmental and societal markers of poverty including maternal education, access to health services and inequality [21].

Immunologic priming from repeated GAS exposure is thought to determine the age of ARF onset, which is greatest between 5 and 15 years [22]. The abnormal host immune response occurs in an estimated 0.3–3% of people following GAS infection [23]. ARF has diverse manifestations, usually a febrile illness with arthritis, carditis and/or cutaneous features (erythema marginatum or subcutaneous nodules). Sydenham’s Chorea occurs in up to 30% of cases (generally 6–9 weeks after infection [24]) and carditis (chiefly valvulitis) in over half of ARF patients [25]. Diagnosis of ARF is made using the syndromic Jones Criteria updated periodically under the auspices of the American Heart Association, most recently in 2015 [25].

The majority of ARF symptoms resolve spontaneously over weeks to months. However, valvular damage persists in an estimated 60% of carditis cases, chiefly affecting the mitral valve [12]. Early valvular damage is characterised by annular dilatation, chordal elongation leading to increased leaflet tip mobility of the mitral valve and associated with mild-moderate regurgitation. These early abnormalities are typically asymptomatic though detectable on echocardiography. This latent phase of the disease is amenable to screening, historically through auscultation and in recent years through echocardiographic studies [26, 27].

**Fig. 1:**
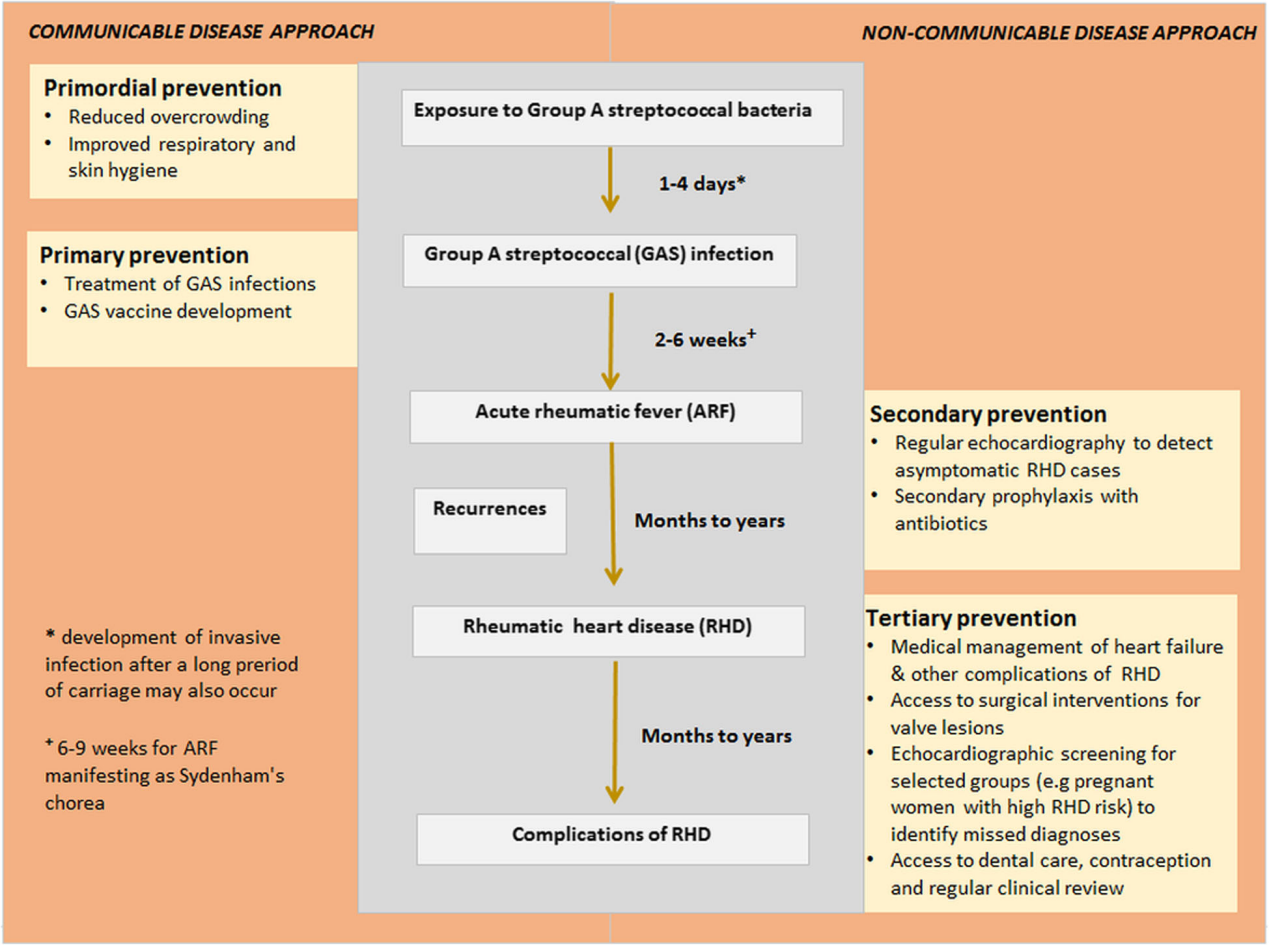
Rheumatic heart disease: disease development and progression. Source: Authors’ compilation

Individuals with a demonstrated predilection for ARF are at risk of recurrent episodes after subsequent GAS infections (Fig. 1). These recurrences drive development and progression of RHD, from initial valvular regurgitation through to stenosis, sometimes complicated by atrial fibrillation, left ventricular failure, secondary involvement of right-sided valves, and infective endocarditis. Females frequently progress from ARF to RHD during their childbearing years; RHD can cause sudden and severe cardiac failure during the perinatal period.

### RHD epidemiology

RHD affects 33.4 million people globally and causes 347,000 deaths annually [28]; 80% of ARF cases occur in LMIC [29]. The REMEDY study [4] conducted in 14 LMICs highlighted the high burden of RHD on young people (particularly women) where access to quality secondary and tertiary prevention services is poor. Indigenous populations of high income countries like Australia [30], New Zealand [31] and Canada [32] have some of the highest documented rates.

The decline of cases in resource-rich settings was associated with reduced crowding, improved socioeconomic environments, access to health care and the widespread availability of penicillin [33]. RHD prevention and treatment approaches were developed and implemented in the 1940s–1960s by clinical researchers in the United States and Europe [34, 35], providing a template for RHD control programs and clinical care worldwide. This foundation has been built on by ongoing basic science studies, implementation research and RHD control programs [34].

### Levels of disease prevention

**Fig. 2:**
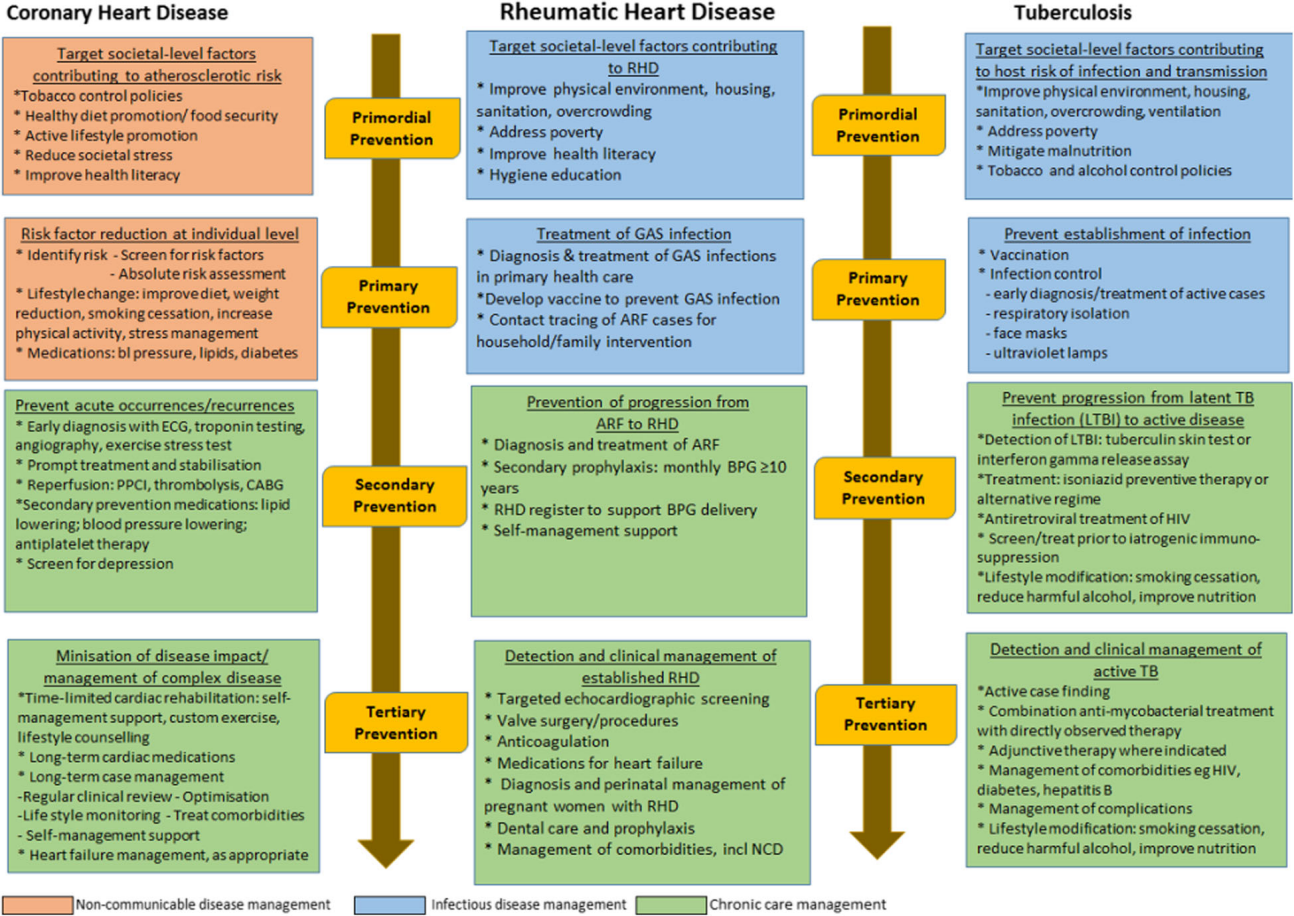
Comparison of strategies to prevent and control rheumatic heart disease, coronary heart disease and tuberculosis. Source: Authors’ compilation

The aetiologic pathway of RHD provides scope for a broad range of disease control strategies (Figs. 1 and 2). These can be considered within the four levels of the prevention framework: *primordial* - addressing the environmental, socio-economic [36, 37], behavioural and cultural factors underpinning disease incidence; *primary* - preventing disease acquisition; *secondary* – interrupting the disease process or preventing complications arising from established disease; and *tertiary* – managing disease to limit consequences. This framework [7, 38] provides a structure for comparing the management strategies relevant to both infectious disease and NCD. The infectious origins of RHD place it between the communicable and NCD ends of the spectrum, providing a novel lens to explore lessons from both sectors.

CHD is the leading cause of human deaths globally [2]. Major successes at all levels of prevention have resulted in a global decrease in CHD-related mortality in the last 20–30 years [39]. However, these improvements have disproportionally benefited high-income settings. CHD control activities developed and refined in HICs [40–42] – including restrictions on smoking areas, plain packaging of tobacco and food labelling – are yet to be broadly adopted in lower-resource settings.

TB is a disease for which evidence-based preventative strategies at all four public health levels are clearly defined. The global importance of TB over many centuries [43] has meant that vertical public health strategies for TB are arguably the best-developed of any control programs. TB demonstrates many important parallels with RHD, including the populations at risk, the absence of a highly sensitive or specific diagnostic test and the challenges of treatment.

The ubiquity of CHD means that it is core business of primary care services worldwide. In contrast, RHD and TB tend to be managed in dedicated vertical programs. The applicability of successes in CHD and TB control to disease prevention strategies for RHD are explored below (Fig. 2).

#### Primordial prevention

Primordial prevention of RHD is predicated on reducing exposure to GAS and GAS infections (Fig. 2). The strongest evidence for a causal association between a primordial (socioeconomic) determinant and RHD risk is for household or bedroom crowding. There have been at least 50 studies over several decades examining the association between crowding and either GAS infection, ARF or RHD. These are mostly of limited quality but collectively, supporting an association between crowding and ARF risk. Examples include the following. In an ecological study undertaken in New Zealand, Jaine et al. identified that the rate ratio of ARF cases was significantly associated with crowding quintile, according to population-level data [44]. In Wannamaker’s pivotal military barracks investigations published in 1954, decreasing distance of one’s bed from a case of GAS infection was identified as a risk factor for GAS acquisition, and rate of acquisition of GAS was also associated with the number of GAS carriers in a barrack group [45]. In a case-control study of 148 ARF cases and 444 controls from Serbia investigating the influence of socioeconomic factors on the occurrence of ARF, smaller available living space (<5 m2), having ≥2 persons per room or have ≥2 persons per bed were all significantly more common among ARF cases than controls [46]. In a more recently Australian study, the number of cases of pyoderma (mostly due to streptococci) per household was directly proportional to the number of people per bedroom in one Aboriginal community [47].

There are limited examples of primordial-level interventions in practice, since strategies to alleviate the elements of poverty that drive RHD are a global challenge. In NZ children with ARF and their families are prioritised for higher quality or larger public housing. NZ Guidelines [48, 49] and draft guidelines under review in Australia by the Communicable Disease Network of Australia, recommend children are educated about respiratory hygiene to reduce GAS spread. In Australia, GAS skin infections (impetigo) also appear to be an antecedent of ARF, hence research activities in some jurisdictions have focused on provision of ‘health hardware’, including running water and soap to reduce skin sores and ARF [50] and other initiatives incorporating community participation in household clean-ups to improve hygiene [51]. Research projects co-designed by researchers and community residents are underway in Northern Australia, aiming to help Aboriginal community members to develop durable, locally-applicable strategies to control GAS transmission and hence, RHD risk [52]. As yet no conclusive evidence is available about how to use primordial interventions as a mechanism for developing a control strategy.

##### How can CHD and TB primordial control strategies be informative for RHD?

CHD demonstrates the value of population-level interventions for cardiac disease control. For instance, there is recognition of the need to use legislative change at jurisdictional or national levels for CHD primordial prevention, rather than reliance on individual-level behaviour change. Such approaches [40–42], for example, compulsory food labelling and tobacco taxation, have successfully reduced rates of CHD. Health promoting legislation may provide inspiration for comparable strategies for RHD, for instance enforcement of minimum housing standards (see example of trial Warrant of Fitness for rental houses [53]) and maximum occupancy rates. Reduced GAS, ARF and RHD would be a long-term benefit of ongoing efforts to improve living standards in deprived communities.

Primordial preventive strategies for TB are similarly instructive for RHD since these epidemics overlap geographically. Over-crowding is a proven facilitator of both GAS and *M. tuberculosis* transmission. The evidence associating TB with poverty has made bold political commitments possible. In particular, the ambitious End TB Strategy calls for poverty alleviation as a TB control strategy [54]. Further quantifying the association between RHD and poverty could yield similar results. These calls could help to amplify the message; advocacy for primordial-level interventions would achieve mutually beneficial outcomes across multiple disease endpoints.

#### Primary prevention

Primary prevention of RHD comprises antibiotic therapy for GAS infections to prevent an initial attack of ARF [55], where administration within 9 days of pharyngitis onset can reduce ARF occurrence by up to 80% [56]. Evidence from a large-scale program to improve access to sore throat treatment in NZ [57] suggests that ARF incidence can be reduced through a multi-pronged approach has the potential to be cost-effective in high risk populations [58]. Sore throat treatment was a key component of broader, comprehensive ARF/RHD control strategies in Cuba [59] and Costa Rica [60]. Access to either laboratory culture facilities or rapid antigen detection tests to confirm GAS pharyngitis remains challenging in many LMICs, leading some to recommend either imperfect clinical prediction rules or alternatively a ‘treat-all’ approach [61]. In high resource settings with a low incidence of ARF, concerns about antibiotic overuse have appropriately curtailed routine antibiotic treatment of pharyngitis. Advances in GAS vaccine development have placed vaccination on the agenda as a future opportunity for primary prevention [62–65].

##### How can CHD and TB primary control strategies be informative for RHD?

RHD prevention strategies can draw on CHD approaches by developing and popularising decision support tools [66] for the identification and treatment of GAS infections in high risk populations. Although clinical decision rules have been developed for assessing people with sore throats and some evaluated in LMIC [67, 68], further validation and optimisation is required. Partnering with NCD health promotion teams working with youth, sharing networks and communication platforms, can also potentially benefit RHD primary prevention if prevention messages are clearly articulated. Case management and contact tracing is standard of care in TB but has received relatively little attention to the management of GAS infections. In particular, focusing on other household members of ARF cases may direct resources towards people exposed to similar environmental risks, GAS strains and genetic predisposition.

#### Secondary prevention

The cornerstone of RHD control internationally is secondary prevention with long-term depot administration of penicillin, or daily oral antibiotic in individuals who are penicillin allergic [69]. In the absence of a vaccine for GAS, secondary prevention has been determined to be the most cost-effective RHD control strategy [70]. Secondary prevention comprises administration of intramuscular, long-acting penicillin every 28 days for individuals with a history of ARF (or documented RHD) to prevent ARF recurrences [55] thereby reducing cumulative valve damage. International guidelines recommend benzathine penicillin G (BPG) as the first line therapy, injected intramuscularly every four weeks [71]. Secondary prophylaxis continues from the first ARF diagnosis until the period of highest clinical risk has passed. Australian [49], NZ [72] and WHO guidelines [71] recommend prophylaxis for a minimum of ten years following last ARF episode or until 21 years of age.

Patients with severe RHD may need lifelong prophylaxis, implying a long-term burden of disability and treatment. The challenge of delivering onerous secondary prophylaxis prompted the development of RHD registers [73]. Although now rare in high income countries, the register-based model has been adapted in low resource settings with increasing standardisation of terms and clinical data collection. New register-based programs are expanding, including examples from Uganda [74], Fiji [75] and East Timor [76]. Register-based secondary prophylaxis remains a backbone of broad RHD control programs, generally considered cost effective [32, 77] although not fully evaluated in very low resource settings.

Echocardiographic screening to identify asymptomatic RHD has provided new impetus for secondary prophylaxis. Echocardiography has replaced auscultation as a screening tool, providing a more sensitive and specific test [78]. The development of echocardiographic diagnostic guidelines for screening has standardised interpretation and reporting of results [79]. Although echocardiographic screening is broadly accepted as an epidemiologic research tool to better quantify the RHD burden [80], the role of echo to identify asymptomatic valve lesions and deliver early intervention is less clear in the absence of good knowledge of the natural history of disease progression [78]. In theory, the use of echocardiographic screening for clinical benefit would be an augmentation of secondary prevention; also, detection in women of childbearing age would permit appropriate planning for pregnancy and childbirth. This is potentially feasible in RHD-endemic populations of HICs where the majority of symptomatic cases have already been detected. In LMICs where RHD is generally more advanced when diagnosed [4], echocardiographic screening detects a higher proportion of symptomatic cases. The disease-altering benefit of secondary prophylaxis in advanced valvular RHD is smaller than in asymptomatic cases as interventions shift from secondary prophylaxis to management of heart failure and surgical options. Nevertheless, screening in these settings identifies cases at a less progressed stage than self-presentation and provides the only means to identify cases at a mild stage when SP has the greatest likelihood of improving outcomes, thus still providing benefit. In reality, the utility of screening programs for clinical benefit is subject to ongoing debate but may be considered as ways of identifying cases for both secondary and tertiary interventions.

##### How can CHD and TB secondary control strategies be informative for RHD?

Strategies to improve secondary prophylaxis of ARF/RHD can benefit from chronic care management approaches characteristic of NCD such as CHD where long-term medication is needed. Indeed, the term ‘secondary prevention’ in the CHD field [81, 82] usually extends to health care that aims to prevent acute coronary events and recurrences as well as treatment of their complications, requiring the management of long-term complex disease [66, 81, 83]. Smartphones and apps for diagnosing, monitoring, reminding and communicating with patients are well-developed in the cardiac and chronic diseases field [84, 85] and can be incorporated into RHD control activities, as is being done in Australia (https://www.rhdaustralia.org.au/resources). Modern management approaches to NCDs commencing in childhood, such as type 1 diabetes and cystic fibrosis, are informative for RHD; the concept of transition care [86], providing a framework to support children through adolescence and to adulthood, is increasingly used for chronic paediatric conditions, and has application in the context of RHD. As patients age, comorbid heart diseases necessitate an integrated approach to addressing both ischaemic and valvular heart disease [87, 88].

Communicable diseases other than RHD can similarly benefit from chronic care approaches; while this is now well-recognised in HIV management, secondary prevention of TB (i.e. treatment of latent TB infection) can also be usefully informed by chronic disease management approaches, for the period of time (usually 6 months but potentially much longer [89]) that such treatment is required.

#### Tertiary prevention

Tertiary interventions for RHD include medical management of heart failure, operative management of valve lesions and treatment for the consequences of RHD, including stroke, infective endocarditis and arrhythmia. Opportunities for operative intervention span catheter-based balloon valvotomy, open commissurotomy, valve repair or replacement. Access to these services is severely curtailed by the gross economic and geographic disadvantage in endemic RHD settings with minimal tertiary cardiac services. A number of programs aim to improve access to advanced cardiac services in RHD endemic, low-resource settings. These initiatives often involve reciprocal training of surgeons, visiting surgical teams and the development of regional centres of excellence [90].

##### How can CHD and TB tertiary control strategies be informative for RHD?

Infrastructure and human resource needs for the management of heart failure and other heart diseases converge in the tertiary setting. These needs are largely independent of aetiology, allowing reintegration of RHD from a vertical register-based program into the broader health system. This is exemplified by work in Rwanda to improve management of all-cause heart failure, including cardiomyopathy, congenital heart disease, CHD and RHD [91]. Similarly, developing cardiac surgical capacity is a shared goal of CHD and RHD, including intermediate steps in access to echocardiography, intensive care units and cardiac catheterisation facilities.

TB control strategies have particular relevance for RHD because of the overlapping socioeconomic risk factors (and hence, affected populations), and the relevance of approaches that emphasise patient health literacy and engagement with the health system. Recent papers have reported successes of community-driven models of care to provide TB knowledge as well as accessible, culturally-appropriate management. This includes the use of ‘care groups’ in Mozambique [92], and ‘health extension workers’ in Ethiopia [93]. Both are examples of task-shifting - that is, investing community members with knowledge and responsibility instead of creating a system reliant on few trained healthcare providers. These approaches are highly applicable to RHD and such models of care need to be tested in high RHD-burden settings. Further, the global requirement to adhere to mandatory reporting of TB key performance indicators can also be informative for RHD. As with TB, the use of standardised treatments and increased opportunistic screening for NCD risk factors, provision of contraception for women and diagnosis of comorbidities can greatly improve outcomes for RHD patients.

### Chronic disease concepts: Application to RHD and TB

Chronic care paradigms are increasingly relevant to chronic diseases with an infectious aetiology as life-prolonging treatment options emerge. Even in the absence of established RHD, an individual who has experienced ARF has a ‘chronic’ condition, given the requirement for a decade of engagement with the healthcare system for long-term medication and regular reviews [49]. Thus control measures at secondary and tertiary levels require a chronic disease management approach (Fig. 2, green highlights).

Similarly, despite TB being widely considered a disease of discrete, six-month duration, chronic ramifications persist after microbial cure. These include impaired lung function and quality of life, residual respiratory symptoms, chronic obstructive pulmonary disease and bronchiectasis [94, 95]. Additionally, many people with TB have chronic comorbidities requiring attention during TB treatment [96, 97]. TB patients would therefore also benefit from ongoing management guided by chronic care approaches [98]. Such approaches are accorded low priority in limited-resource settings, since chronic complications after TB are under-recognised and do not pose a substantial public health threat. Better recognition of chronic pulmonary sequelae post-TB are required to ensure appropriate referral to generalists or pulmonologists to provide chronic care management. This discussion illustrates points of relevance to RHD: the value of drawing on NCD approaches in managing diseases of infectious aetiology and the requirement for vertical programs to have strategies to ensure appropriate hand-over of care individuals with chronic sequelae of their infection.

### Whither RHD: Vertical, horizontal and diagonal programs?

#### Vertical versus horizontal

The advantages of vertical programs– their specific disease focus, use of specialist staff, dedicated resources and focused objectives –generally do not extend to NCD services which are usually part of (horizontal) mainstream services. Where both infectious diseases and NCD impart high burden in a single population, the need for co-ordinating resources and systems is now being recognised [3, 97, 99].

The RHD sector intersects with many specialities and patient groups, providing opportunities for increased integration with both vertical programs (like TB and HIV/AIDS) as well as mainstream primary health services (like maternal and child health, NCD services and adolescent health). A stark example of the need for integration can be seen in the REMEDY study of RHD hospital patients in 12 LMICs, where only 4% of the women with RHD of childbearing age were receiving contraception [4].

#### Diagonal

A ‘diagonal’ approach is increasingly promoted as a way to strengthen health systems, harnessing aspects of successful and well-resourced vertical programs to support other health services [100, 101]. This calls for disciplinary interaction and alignment [99] by looking for opportunities to combine prevention, screening, and treatment programmes.

Diagonal approaches often start with vertical programs [101–103], with incremental integration of subgroups of patients with overlapping conditions, expanding to other patient groups as services consolidate and service partnerships develop [104]. There are now abundant reports in the literature showing how donor funding, infrastructure, systems and services for HIV/AIDS have been leveraged to improve other generic services like maternal and child health, and NCDs [105–107]. Collaborative frameworks for the care and control of TB and diabetes [97] and TB and HIV [96] grew out of the recognition of the interaction between these diseases, suggesting how overlaps between conditions can lead to clinical integration. An important starting point is screening for other diseases in vertical programs as shown in Malawi where TB case-finding was enhanced in HIV patients and TB programs were an entry point for HIV care [96]. In Mexico, MCH services were enhanced by building onto vertical programs in a step-wise fashion [103]. Despite these encouraging case studies, there is a need for more evidence of what works and not [96, 101, 107], while recognising the contextual complexity and that no ‘one-size fits all’ exists [102].

RHD illustrates some of the challenges in operationalising diagonal or integrative models of care, even when strong conceptual links and potential benefits are evident. There are few published examples of integration between vertical programs: a pilot study to explore the utility of screening HIV infected children for RHD has been conducted in Uganda [108] and in Eritrea a pilot program screened pregnant women for RHD [109]. A shift towards integrated, comprehensive care for RHD and other conditions is expected to accelerate – spurred by global efforts to improve universal health coverage and integrated, people-centred health services [110]. Within RHD this shift necessities a common nomenclature for describing integrative activities. A descriptive framework is presented in Table 1, differentiating between adding RHD elements to existing programs or adding other disease elements to RHD programs.

**Table 1:** Activities supporting integration of RHD into horizontal and vertical programs

	Adding RHD elements to existing programs	Adding other disease elements to RHD programs (target PLW*-RHD)
Vertical programs	• Sexual and reproductive health services: provision of secondary prophylaxis, echo screening of targeted high-risk pregnant women • HIV: RHD secondary prophylaxis for people with HIV and RHD co-morbidity & echocardiographic screening of targeted high-risk PLW** HIV • TB: Delivery of secondary prophylaxis through DOTS for people with TB and RHD co-morbidity	• Dental care: enchancing dental services for PLW-RHD to reduce endocarditis • Specialist Telemedicine • NCD* programs: Risk assessment and primary prevention of ischaemic heart disease to tackle co-morbidity for PLW RHD • Infectious diseases: Screening for infectious diseases in PLW-RHD, particularly prior to surgical intervention.
Horizontal programs	• School programs: streptococcal pharyngitis and impetigo screening/treatment, secondary prophylaxis support • Environmental health: education and programs to target streptococcal pharyngitis and impetigo • Case Management: to support adherence & navigate the healthcare system • Medication supply: prioritisation of procurement of benzathine penicillin G. • Primary health care information technology solutions: RHD modules added to electronic/paper health records • Skills/training of primary health care staff	PRIMARY HEALTH CARE SETTING • Delivery of tertiary care in the primary setting: supporting advanced care for complications of RHD, including management of heart failure, arrhythmia and anticoagulation (including dental care and Telemedicine where available). • Lifestyle interventions: smoking, exercise, diet • Risk assessment: for coronary heart disease • Screen for diabetes, chronic kidney disease • Sexual & reproductive services for PLW-RHD • Case management

*NCD Non-communicable disease **PLW: people living with

Using this framework, it is possible to describe a range of early stage activities as integrative approaches. For example, in Australia, completion of training modules on RHD in pregnancy is now a mandated component of midwifery training, demonstrating the addition of RHD elements to existing programs [111]. Nascent programs are also beginning in continental Africa to embed RHD activities into existing maternal health infrastructure. Conversely, training RHD care providers to improve management of non-RHD conditions is integrative – illustrated by recent efforts to improve care of anaphylaxis by primary care providers who administer benzathine penicillin G injections in Zambia [112]. Integration with primary care delivery may also be evident if targeted echocardiographic screening for RHD, currently being tested with regards to sensitivity and specificity when undertaken by non-specialist primary care providers [113–116], becomes more mainstream over time.

**Fig. 3:**
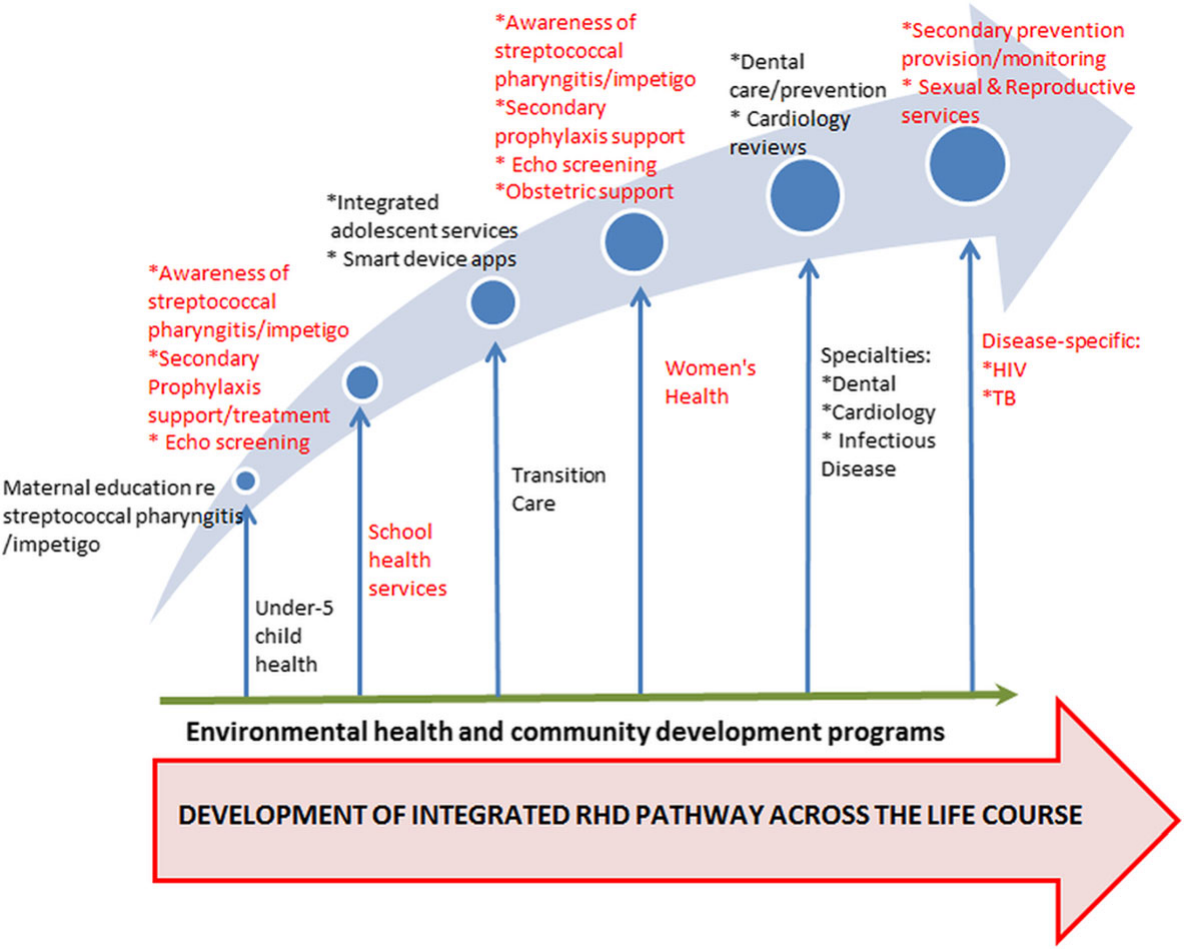
Diagonal approach to harness inputs from vertical programs for rheumatic heart disease: pathway across the life course. Source: Authors’ compilation

Thus inputs from vertical programs can be harnessed for RHD control efforts at various points along the life course (Fig. 3), with the primordial factors required to be addressed at all life stages through synergies with community development and environmental health programs.

### Integrating RHD programs into existing chronic disease initiatives

#### The chronic care model(s)

In the mid-1990s, following a thorough review of the evidence [117], the MacColl Institute for Healthcare Innovation developed the Chronic Care Model (CCM) as a conceptual model for quality improvement in chronic disease management. The CCM is an approach to chronic illness management which aims to improve service delivery by creating an ‘informed, activated patient and a prepared, proactive practice team’. The model outlines themes relevant to the health system (Health Systems, Delivery System Design, Decision Support, Clinical Information Systems) and community (Self-Management Support and Community Linkages) to consider when providing chronic care management in primary care settings. The CCM therefore provides a relevant framework for improving the delivery of secondary prevention of RHD.

This model has been successfully applied in many health care organisational settings in the United States, with research evidence accumulating that system changes consistent with CCM components/principles improve outcomes in chronic diseases [118, 119], ranging from diabetes [120–123] to chronic heart failure [121] and COPD [124, 125]. The model has also been applied to cancer [126], depression [127] and neurology [128], and translated into practical application in the Australian Indigenous primary care setting [129].

The CCM was used as a reference point to develop the Innovative Care for Chronic Conditions (ICCC) Framework of the WHO [8, 130]. The ICCC was developed to ensure applicability in low and medium income countries through greater linkage of the policy environment and community context to health care organisation. Further, an enhanced version of the CCM, the Expanded CCM [131], was proposed to incorporate public health/health promotion concepts and strategies, in particular the social determinants of health [36, 37] which influence not only secondary and tertiary levels of prevention, but impact profoundly on primordial and primary prevention of both infectious disease and NCD. The three members of the CCM-family each have a different emphasis, however all recognise the centrality of good organisational systems for managing long-term conditions.

#### Application of these models to RHD

**Fig. 4:**
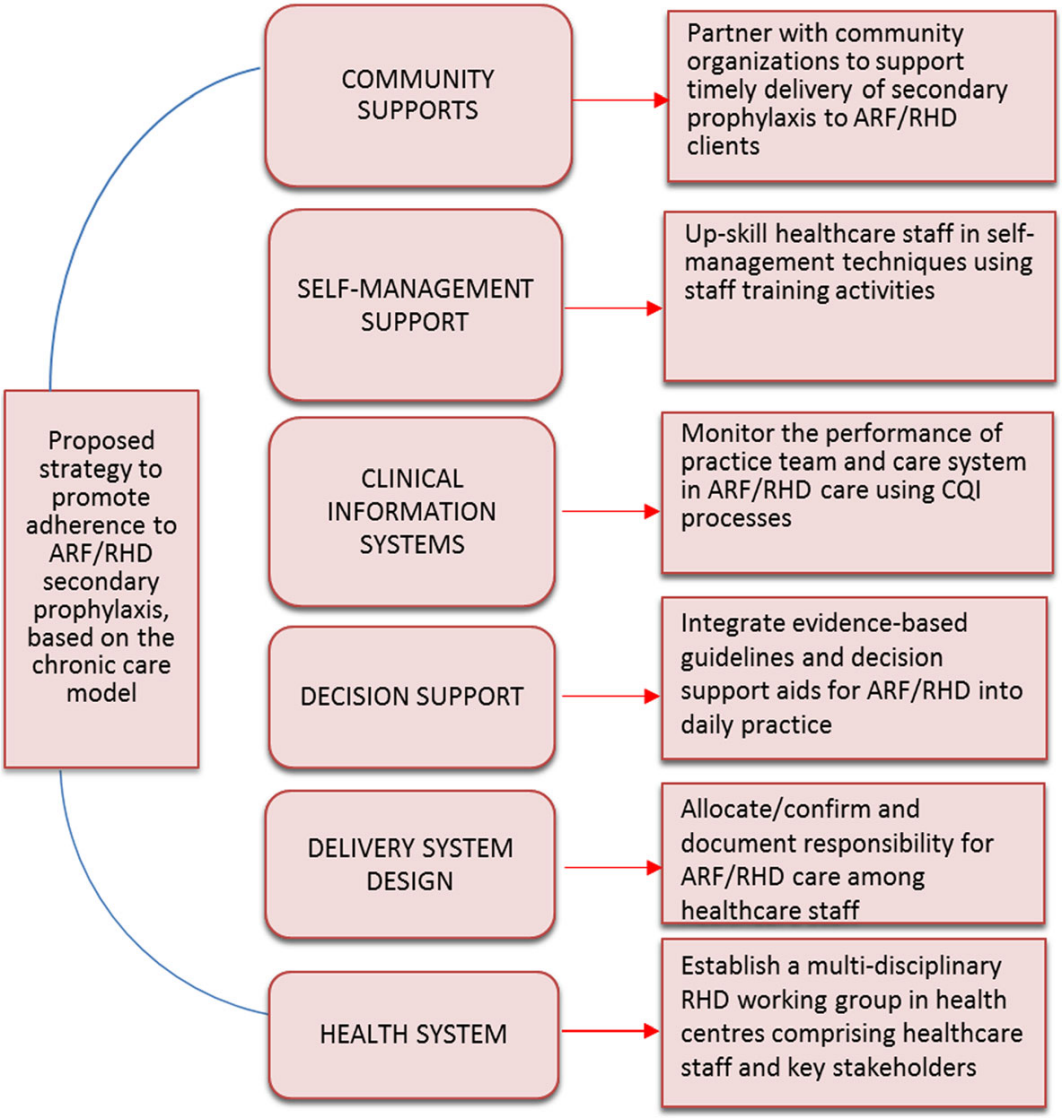
Application of the Chronic Care Model to an intervention to improve ARF/RHD secondary prophylaxis. Source: Compiled by investigators of the Secondary Prophylaxis Trial, not previously published in this form and approved for publication in this article by investigators, including one of the authors (APR)

We propose that the CCM models can be operationalised for RHD by greater integration of secondary and tertiary prevention into existing services [132]. Secondary prophylaxis is the most important investment to make [70] yet adherence to secondary prophylaxis tends to be low internationally. Secondary prevention clearly fulfils the definition of ‘chronic care’, even though it applies to ‘acute’ rheumatic fever. After ARF, chronic care management entails 4-weekly contact with a healthcare provider for administration of secondary prophylaxis (a depot penicillin injection) for a minimum 10-year period, as well as regular medical evaluation. Adherence is challenging since the treatment is a painful injection; healthcare staff often have poor knowledge of ARF and RHD; patient populations are young, often asymptomatic and in some settings, mobile; and health literacy pertaining to the value of injections may be low [133]. Models that have the potential to improve adherence could have important value in this context. In a before-after study implementing a continuous quality improvement (CQI) process using participatory action research, we showed improved delivery of aspects of ARF/RHD care to Aboriginal patients, but a failure to improve adherence to acceptable levels [134]. CCM-based CQI methods applied to NCDs in the same settings (clinics in remote Australian Aboriginal communities) have successfully improved patient outcomes [135]. A randomised community trial is currently underway in a high-burden Australian jurisdiction to determine the impact of systems interventions incorporating the components of the CCM on uptake of regular BPG for the secondary prevention of ARF [133]. Tailored use of the CCM has been developed as the basis for the trial as shown in Fig. 4, using the six CCM themes as the scaffold for primary health care activities to implement within the study intervention. This intervention exemplifies a diagonal approach where primary health care services are augmented through ‘vertical’ support to improve outcomes. The mainstream services provide the infrastructure and context for RHD care to be integrated into other essential services, providing better opportunity for all-of-person management.

The CCM also has a particularly good fit with tertiary prevention of RHD. In well-resourced programs, other aspects of chronic care after ARF include regular (e.g. yearly) echocardiography and dental care [49]. Thus the life-long requirement for cardiac review, monitoring of medications (including anticoagulation) and the potential need for surgery means that patient-centred case management approaches can have a significant impact on quality of life and survival. The need for a model of long-term care of RHD patients in LMIC is illustrated by a recent global registry study [136] showing 17% two-year mortality among 3343 children and adults with RHD from LMIC. The financial expenditure of tertiary-level interventions is wasted if continuity of care for these patients is not provided in the community. In many low resource settings, existing primary care services are very weak and/or geared towards acute, rather than ongoing care. Adoption of the CCM for RHD patients can support and promote strengthening of the primary care system, and better integration with tertiary services.

#### Application of the CCM to CHD and TB

CHD, particularly its association with diabetes, is a common focus of the CCM [121, 122]. The requirement for life-long adherence to cardiac medications and lifestyle modifications as well as the need for treatment of comorbidities means that the patient-centred CCM provides a useful framework for long-term management of CHD. In the management of TB (and other infections), some components of the CCM may be utilised during the antibiotic treatment phase, but not beyond, despite the potential benefits after microbiological cure. Well-functional TB control programs have specific public health mechanisms to support care and reduce patient default, provide patient education, provide decision support to practitioners through written national TB treatment policies, and good data collection to provide feedback nationally and to the WHO. While these structures are nominally in place internationally, there is always scope for improvement in the quality of implementation, to ensure the desired outcome of ‘informed, activated patients and communities’ and ‘prepared, pro-active practice teams and community partners’ as described in the chronic care model [131]. Two themes of the CCM emphasise the importance of patient and community engagement – the ‘self-management support; and ‘community engagement’ themes. New community-driven approaches to TB control, mentioned above [92, 93] provide informative strategies that could be used to by RHD control programs to address these themes of the CCM.

### Health system considerations

Scope to ‘diagonalise’ RHD services through integration of horizontal and vertical service components, and provide quality long-term case management is heavily dependent on the capacity of the underlying health system. The practical, financial and capacity limitations on disease control often reflect fragile health systems of LMICs. Therefore, health system strengthening can be considered a critical component of integrative care for RHD, TB and HIV. Consideration of the building blocks within the WHO Health Systems Framework shows how these apply in the RHD context.

#### Leadership/governance

The burden of RHD is grounded in social and economic determinants of health, necessitating a political and government response. In some places, RHD has become an explicitly political issue. For example, in New Zealand, rheumatic fever rates have been a feature of two election cycles and major government investment has reduced the rate of ARF by 23 % [137, 138]. In 2015, the Addis Ababa Communique on ARF and RHD control was adopted by Heads of States of African Union member countries [139]. However, in the majority of LMIC endemic settings government and governance responses are thinly spread and often overwhelmed by competing demands. Thus there may be scope to frame RHD as a sentinel disease of inequality, facilitating strategic investment in primary care and equity advancing interventions [140]. Political interest may be further channelled into global initiatives, including growing efforts towards universal health care (UHC) [141] and a pending discussion of RHD control at the World Health Assembly 2018 [6].

#### Health care financing

Financing for RHD interventions is often skewed towards investments in costly tertiary intervention. In particular, a large number of RHD endemic LMIC send patients overseas for expensive valve surgery [142], sometimes with poor outcomes. A health systems strengthening approach would see government funds disbursed earlier in the aetiologic pathway with greater potential for prevention and primary care settings where multiple other diseases can also be addressed. However, in some settings early interventions (primary/secondary prevention) incur out-of-pocket cost, reducing access and thus efficacy of the interventions [143, 144]. RHD is a good example of how investing in UHC has the strong potential to be cost saving. Spanning the infectious disease – chronic disease continuum RHD can help illustrate to policy makers that providing appropriate antibiotics in primary care can save expensive international heart surgery. Illustrative narratives are influential for decision makers [145] and RHD offers compelling vignettes to address financing issues.

#### Health workforce

Diagnosis and management of RHD is human-resource intensive at all levels, including for early interventions of pharyngitis management, ARF diagnosis and secondary prophylaxis delivery. Increased human resources for health are needed globally. In the interim, task shifting [115] and task sharing may be opportunities to ameliorate shortages. In particular, given the shortage of expert operators for cardiac ultrasound, training programmes are under development for non-expert operators to undertake limited echo views for screening purposes [113, 116]. In addition to this kind of technological solution ongoing work is needed to train and support nurses and community health workers to diagnose and manage strep pharyngitis, ARF and RHD. Simple, standardised clinical guidelines are a critical component of this support and are urgently needed for RHD control.

#### Medical products, technologies

Access to essential medicines and essential technologies for RHD care continue to be major global barriers. In particular, the critical injectable antibiotic, benzathine penicillin G (BPG) has been subject to global shortages over a number of years [146]. An old, off-patent, affordable antibiotic which has been on the WHO Essential Medicines List since inception, BPG should be readily available. The difficulties in procurement and use of BPG exemplify access to medicines issues globally and are an appealing target for government and multi-state intervention. The WHO Package of Essential NCD Interventions for Primary Health Care (PEN) identifies essential technologies relevant to RHD, particularly stethoscopes, scales, and blood pressure monitoring. [147] ECG capacity is identified in PEN as an essential technology ‘where resources permit’. Supporting access and supported use of the basic clinical resources in primary care should be a shared priority. Although expanding access to echocardiography for diagnosis and clinical review offers considerable scope for improve quality of care for RHD and other cardiac conditions, the role of population level echocardiographic screening remains under investigation [148]. The cost and training required to take advantage of portable and hand-held imaging technology remain significant barriers to health systems, but could revolutionise diagnosis, triaging and management of ARF/RHD in the future.

#### Information and research

Many LMICs have insufficient contemporary data on ARF/RHD occurrence to inform health system responses [28, 149]– both at the policy and programmatic levels. Concerted efforts are needed to fill information gaps, in particular recording of longitudinal data to inform ARF/RHD care. Classically, RHD registers have been used to record clinical features and adherence with secondary prophylaxis. As electronic health information systems increase in endemic settings, there is increased scope to integrate register-based care [150]. In addition to improving case management, this may offer new opportunities to understand disease progression and mortality outcomes of RHD. Documented efforts to integrate RHD registers into routine care information systems are needed to identify best practice. This exemplifies the need for ongoing implementation research science in RHD control.

#### Service delivery

The unanswered challenge in RHD control is identifying service delivery models which are acceptable to populations at risk, to deliver effective interventions that are sustainable in the face of competing priorities. The incremental integration, diagonal approaches and health system strengthening recommended earlier in this paper are likely to be components of identifying feasible models. These models need to be evaluated to provide evidence-based recommendations for policy and program design.

## Conclusion

The continuing burden of RHD in LMICs, as well as in vulnerable communities in high-income countries, requires innovative solutions drawing on established models of care which have proven beneficial in other settings.

Three key points mean that RHD prevention and management must leverage off other well-established models of care in the infectious diseases and NCD realms. Firstly, RHD causes an immense illness burden, including heart failure, stroke and death in children and young adults. Secondly, RHD is a unique disease – a cardiac condition for which tertiary management resembles CHD treatment, yet primordial, primary and secondary prevention is built on the fact that it is a communicable infection which thrives in settings of poverty. Thirdly, the settings bearing the heaviest RHD burden are generally those which have least capacity for services and research.

While the body of ARF/RHD literature is now growing rapidly, such that there are increasing RHD-specific data available to guide program development, the cross-fertilisation of ideas from the comparative domains which we describe here can provide an important means of accelerating progress. There is a need to develop and further refine innovative, location-specific systems-level interventions to allow successful implementation of treatments that have been known to work since the 1950s. Specifically, policies to adopt the CCM framework for the secondary and tertiary prevention of RHD in settings with limited resources, in conjunction with strengthening of systems through integration/linkages with other well-performing and resourced services has the potential to significantly reduce the burden of RHD globally. More funding for implementation research into different models of care in LMIC settings is required to provide a strong evidence base for RHD policy and practice.

## Abbreviations

AIDS: Acquired immune deficiency syndrome
ARF: Acute rheumatic fever
BPG: Benzathine penicillin G
CCM: Chronic care model
CHD: Coronary heart disease
GAS: Group A Streptococcus
HIV: Human immunodeficiency virus
ICCC: Innovative care for chronic disease
LMIC: Lower and middle income countries
NCD: Non-communicable diseases
RHD: Rheumatic heart disease
TB: Tuberculosis
